# High-Resolution Mapping of the Novel Early Leaf Senescence Gene *Els2* in Common Wheat

**DOI:** 10.3390/plants9060698

**Published:** 2020-05-30

**Authors:** Na Wang, Yanzhou Xie, Yingzhuang Li, Shengnan Wu, Shuxian Li, Yu Guo, Chengshe Wang

**Affiliations:** State Key Laboratory of Crop Stress Biology for Arid Areas, College of Agronomy, Northwest A&F University, Yangling 712100, China; wangnawheat@163.com (N.W.); lyz13077031@163.com (Y.L.); wushengnan666@163.com (S.W.); lishuxian0706@163.com (S.L.); gy15598128707@163.com (Y.G.)

**Keywords:** wheat (*Triticum aestivum* L.), early leaf senescence, mutant, single-nucleotide polymorphisms (SNPs), KASP markers, fine mapping

## Abstract

Early leaf senescence negatively impacts the grain yield in wheat (*Triticum aestivum* L.). Induced mutants provide an important resource for mapping and cloning of genes for early leaf senescence. In our previous study, *Els2*, a single incomplete dominance gene, that caused early leaf senescence phenotype in the wheat mutant LF2099, had been mapped on the long arm of chromosome 2B. The objective of this study was to develop molecular markers tightly linked to the *Els2* gene and construct a high-resolution map surrounding the *Els2* gene. Three tightly linked single-nucleotide polymorphism (SNP) markers were obtained from the Illumina Wheat 90K iSelect SNP genotyping array and converted to Kompetitive allele-specific polymerase chain reaction (KASP) markers. To saturate the *Els2* region, the Axiom® Wheat 660K SNP array was used to screen bulked extreme phenotype DNA pools, and 9 KASP markers were developed. For fine mapping of the *Els2* gene, these KASP markers and previously identified polymorphic markers were analyzed in a large F_2_ population of the LF2099 × Chinese Spring cross. The *Els2* gene was located in a 0.24-cM genetic region flanked by the KASP markers AX-111643885 and AX-111128667, which corresponded to a physical interval of 1.61 Mb in the Chinese Spring chromosome 2BL containing 27 predicted genes with high confidence. The study laid a foundation for a map-based clone of the *Els2* gene controlling the mutation phenotype and revealing the molecular regulatory mechanism of wheat leaf senescence.

## 1. Introduction

Wheat is one of the most widely grown crops in the world, providing more than 20% of the calories and protein consumed by the human population [1]. Maintaining a high and stable yield of wheat cultivars is a huge challenge for both wheat breeders and researchers. Leaves are the main photosynthetic organ of wheat plants. A previous study showed that the photosynthetically active flag leaf can contribute 30%–50% of the assimilates for grain filling and yield [2]. The early senescence of functional leaves significantly reduces photosynthetic time and efficiency, seriously affecting grain yield and quality in wheat [3]. Therefore, discovering the genes responsible for early leaf senescence is important not only for the elucidation of the early leaf senescence mechanism, but also in terms of its applications in the development of wheat germplasms and cultivars.

Plant leaf senescence involves a complex and highly regulated process with the coordinated actions of multiple pathways [4,5]. In recent years, researchers have elucidated the molecular mechanisms of leaf senescence in model plants, and isolated and characterized several senescence-associated genes that are involved in chlorophyll (Chl) degradation [6,7,8,9], reactive oxygen species (ROS) scavenging [10,11,12], carbon and nitrogen imbalances [13,14,15], and hormone responses [16,17,18,19,20]. However, limited information is available about leaf senescence regulation in wheat. Several studies focused on the transcriptome of wheat leaf senescence [21,22,23,24]. These studies have provided global and potential gene dynamic expression changes and signalling pathways for leaf senescence regulation in wheat, but few genes involved in the regulation of wheat leaf senescence have been identified. Several early leaf senescence mutant genes in wheat were mapped using molecular markers [3,25,26]; however, our understanding of the molecular mechanism of the leaf senescence trait in wheat is limited because none of these genes have been fine mapped and cloned. 

The induced mutants have also become important for forward genetic analysis, where genes underlying mutant traits can be mapped using molecular markers [25]. Various classes of molecular markers, particularly simple sequence repeat (SSR) markers, have been widely used to identify mutant genes in wheat [27,28,29,30,31]. However, it is difficult to develop a high-resolution genetic linkage map based only on SSR markers because of their limited number and scattered distribution on wheat chromosomes. Single-nucleotide polymorphisms (SNPs) are the most abundant type of molecular marker in plant, and a large number of SNPs have been discovered in wheat with the improvement of sequencing techniques [32,33,34,35,36]. At present, several kinds of wheat SNP arrays have been reported, such as the Illumina Wheat 9K iSelect SNP array [37], the Illumina Wheat 90K iSelect SNP genotyping array [38], the Axiom® HD Wheat genotyping (820K) array [39], the Wheat Breeders’ 35K Axiom array [40], 660K [34] and the Wheat 55K SNP array [41]. The 660K SNP array [42] has been effectively applied to develop Kompetitive allele-specific polymerase chain reaction (KASP) markers for saturation mapping of the target genes. Examples include the molecular mapping of the dwarfing genes *Rht12* [43] and *Rht24* [44], stripe rust resistances *Yr26* [45], *YrH62* [46] and *Qyrnap.nwafu-2BS* [47], and the chlorina mutant gene *cn-A1* [48].

We previously reported the first case of the early leaf senescence mutant in wheat, which was controlled by the single incomplete dominant gene *Els2* [49]. The *Els2* gene was preliminarily mapped on chromosome arm 2BL in a 0.95 cM genetic interval between intron polymorphism (IP) markers 2BIP09 and 2BIP14 [49]. The objectives of this study were (1) to develop molecular markers closely linked to *Els2* using the wheat 660K SNP array, and (2) to construct a high-resolution linkage map.

## 2. Results

### 2.1. Development of Kompetitive Allele-Specific Polymerase Chain Reaction (KASP) Markers Based on the 90K Single-Nucleotide Polymorphisms (SNP) Array 

**Figure 1:**
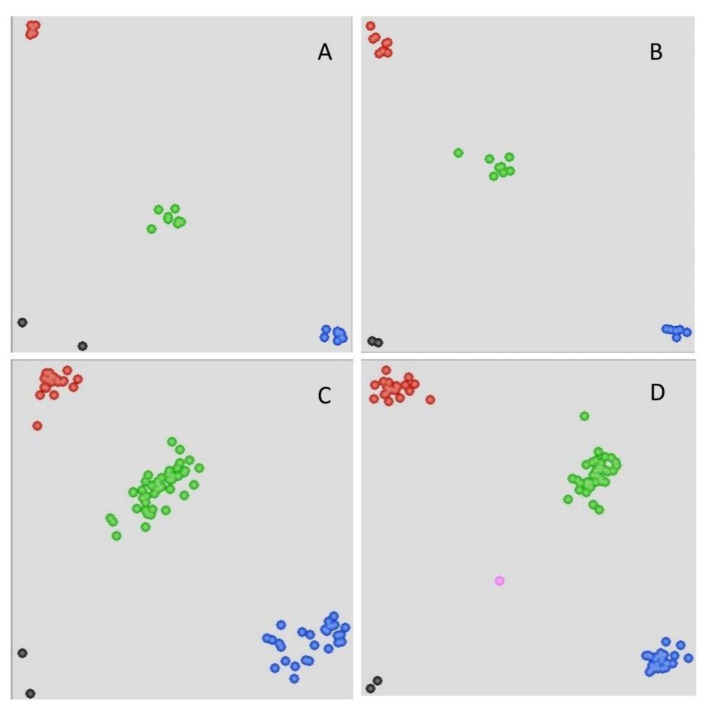
Genotypic plots from selected Kompetitive allele-specific polymerase chain reaction (KASP) assays. The results of the parents and small population using markers (**A**) BS00084417_51 and (**B**) AX-111128667. Results of the F_2_ population using markers (**C**) RFL_Contig4423_529 and (**D**) AX-95682571. The central cluster (green) comprises heterozygous individuals, whereas the clusters near the axes are homozygous for either LF2099 (red) or Chinese Spring (blue). The black dots represent the nontemplate control, and the pink dots represent missing or failed data.

**Figure 2:**
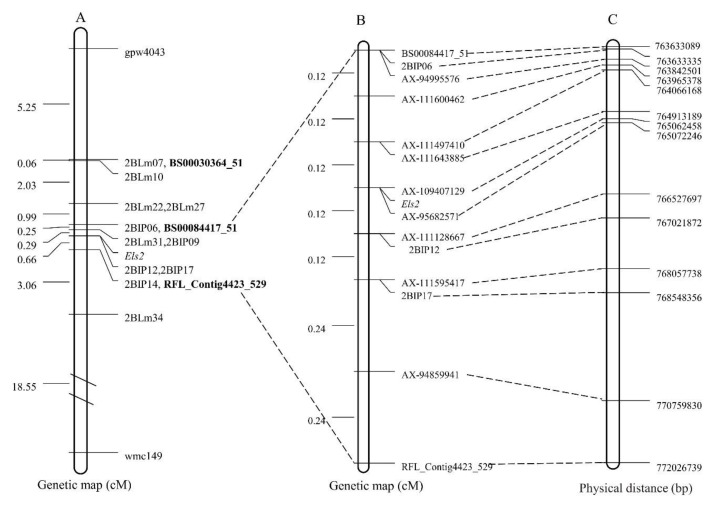
The genetic linkage map and physical map of Chinese Spring genomic sequence fanking the *Els2* gene. (**A**) The preliminary genetic linkage map of the *Els2* gene [49]. (**B**) The high-resolution genetic map of the *Els2* gene. (**C**) The corresponding physical location of the polymorphic linkage markers of the *Els2* gene.

Previously, the early leaf senescence *Els2* gene was mapped to chromosome 2BL using the Illumina wheat 90K SNP array [49]. Given that the SNP array has limitations in large scale population analysis, 15 chromosome-specific SNPs generated from 90K SNP were converted into 11 KASP primer pairs. They were subjected to a polymorphism analysis on the parental cultivars and 20 F_2_ plants (including six homozygous wide type, eight heterozygous, and six homozygous mutant plants). Three polymorphic KASP markers (BS00030364_51, BS00084417_51 and RFL_Contig4423_529) were identified (Figure 1A). By genotyping the previous mapping population consisting of 142 F_2:3_ lines from the LF2099 × Chinese Spring cross [49], all three KASP markers linked to the *Els2* gene (Figure 1C, Table 1). The *Els2* gene was then genetically restricted to a region of 1.1 cM, 0.54 cM from the KASP marker BS00084417_51 and 0.66 cM from the KASP marker RFL_Contig4423_529 (Figure 2A). However, the *Els2* genetic region were not narrowed down by using KASP markers from the 90K SNP array.

### 2.2. Development of KASP Markers Based on the 660K SNP Array 

**Figure 3:**
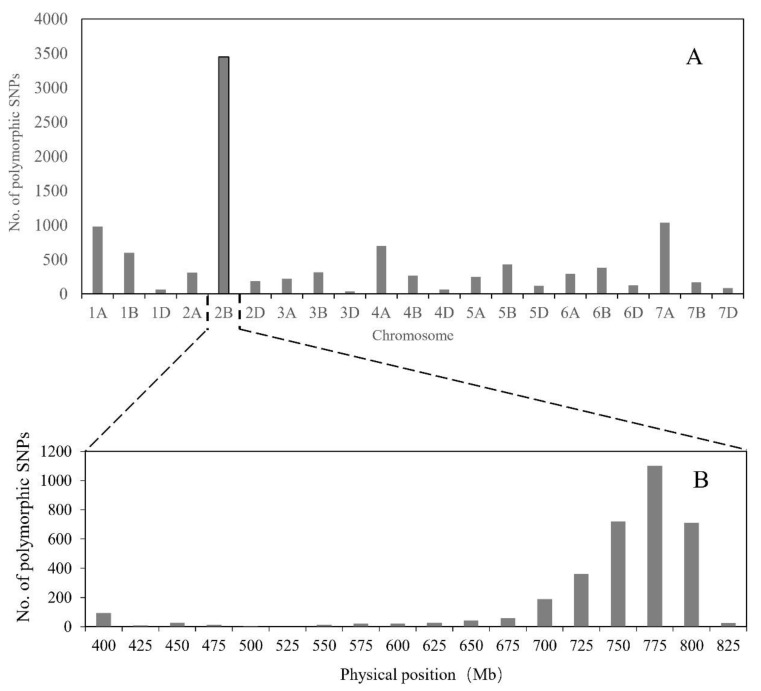
Number of polymorphic single nucleotide polymorphisms (SNP) distributed on different wheat chromosomes (**A**) and distribution of SNP variants on chromosome 2B (**B**).

To saturate marker density in the *Els2* region, we combined bulked segregant analysis (BSA) with 660K SNP arrays. A total of 10,097 SNP loci from the 660K SNP array were polymorphic between the two bulks and were located on 21 chromosomes, of which 3449 were located on chromosome 2B and the others were distributed on the other chromosomes (Figure 3A). These results were consistent with the previous 90K SNP genotyping result [49]. Of the 3449 SNP loci located on 2B, more than 1100 were positioned in the interval 750–775 Mb according to the wheat genome assembly (Figure 3B). Furthermore, two *Els2*-flanking KASP markers were located in the region 750–775 Mb (Figure 2A,C). To develop molecular markers more tightly linked to the *Els2* gene, 25 SNPs located within the interval between BS00084417_51 and RFL_Contig4423_529 (763–772 Mb) were selected for conversion to KASP markers. The results indicated that nine KASP markers were tightly linked to the *Els2* gene (Figure 1B,D, Table 1). 

### 2.3. Construction of a High-Resolution Genetic Linkage Map

To construct a high-resolution genetic linkage map of *Els2*, we developed a large F2 population of 838 plants. The F_2_ population showed three classes of trait distribution, of which 198 were wide type plants, 419 were intermediate plants and 213 were mutant plants. The χ^2^ test shows a separation ratio of 1:2:1 (χ^2^ = 0.53 < χ^2^_0.05,2_ = 5.99), which were consistent with our previous observations [49].

The *Els2*-flanking KASP markers BS00084417_51 and RFL_Contig4423_529 were used to identify recombinants in the F_2_ mapping population of the LF2099 × Chinese Spring cross. Only nine recombinant individuals were identified from 838 F_2_ individuals. Other molecular markers, including the previously reported IP markers 2BIP06, 2BIP12 and 2BIP17 [49], were then analyzed with these recombinants only. The F_2:3_ families produced by selfing the recombinant F_2_ plants were used to further estimate phenotypes. In combination with the marker genotypes and recombinant phenotype, a high-resolution genetic map around the *Els2* locus was eventually constructed (Figure 2B). Among the markers flanking the *Els2* gene, the KASP markers AX-111643885 and AX-111128667 were the most closely linked, at distances of 0.12 and 0.12 cM, respectively. Two KASP markers, AX-109407129 and AX-95682571, co-segregated with the *Els2* gene. 

### 2.4. Physical Mapping and Gene Annotation of the *Els2* Goal Interval

To determine the physical locations of the *Els2*-linked markers, the sequences of all markers anchored in the high-resolution genetic map were used to blast against the Chinese Spring genomic sequence, and the relative physical positions of those markers were generally consistent with the genetic linkage map (Figure 2B,C). The genetic region of the markers AX-111643885 and AX-111128667 corresponded to a 1.61 Mb (764913189–766527697) genomic interval in the Chinese Spring reference genome (Figure 2C) and contained 27 annotated protein-coding genes (Table 2). Among them, one 50S ribosomal protein L14, five cytochrome P450 family proteins, three cysteine proteinases, two serine/threonine-protein kinase, and one RECEPTOR-like protein kinase were identified.

## 3. Discussion

Mapping functional genes using ethyl methanesulfonate (EMS) mutants combined with map-based cloning has been applied in many crops; however, the high-resolution mapping of a mutant gene in wheat may become problematic and tedious without high-density molecular markers. Several approaches have been used to develop high-density molecular markers around the target gene. Comparative genomics analyses are traditional and effective approaches for marker development in a wheat positional cloning project [50]. By applying comparative genomics analyses, we previously developed five new markers linked to the *Els2* gene [49]. Although release of the Chinese Spring whole genomic assembly sequences [51] would reduce the importance of comparative genomics analyses in marker development, it is still an effective method for other species without a reference genome sequence.

The SNP genotyping assays, namely 90K [38] and 660K [42], have opened a new way to undertake a more efficient and faster marker saturation of target genes in wheat [47]. Compared to the 90K SNP chip, the 660K chip has several advantages, such as higher marker density and higher resolution, as well as a better distribution on the chromosomes [42,44]. In the present study, we used the 660K chip to screen candidate markers linked to *Els2* and a significant number of polymorphic SNPs were identified (Figure 3A,B). To improve the efficiency of genotyping individuals, polymorphic SNP markers on 2BL were converted to KASP markers to genotype the F_2_ population. Using this strategy, the *Els2* locus was further narrowed down to a 1.61 Mb region in the Chinese Spring chromosome 2BL containing 27 predicted genes with high confidence (Figure 2B,C, Table 2). It was a clearly effective approach to exploit constantly the updated genome information and high-throughput SNP platforms for high-resolution mapping of a mutant gene in wheat. In addition to the methods used in our study, the combination of the BSA strategy with next-generation sequencing technology (RNA-Seq or exome sequencing) has been used as a mapping strategy that offers the promise of a rapid discovery of genetic markers linked to target genes in wheat [3,52,53].

The *Els2* genomic interval was narrowed down to the 1.61 Mb interval of the Chinese Spring 2BL containing 27 predicted protein-coding genes. Some of the annotated genes were associated with plant early leaf senescence, including the 50S ribosomal protein L14, cytochrome P450 family proteins, cysteine proteinase, serine/threonine-protein kinase, and RECEPTOR-like protein kinase (Table 2). In rice, the gene encoding the 50S ribosomal protein L21 in the chloroplastic precursor 50S ribosomal protein may be responsible for the early senescence mutant *es-t* [54]. Cytochrome P450 CYP89A9, which is in charge of non-fluorescent dioxobilin-type chlorophyll catabolite accumulation, is drawn into the formation of major chlorophyll catabolites during leaf senescence [55]. Cysteine proteases are the most abundant enzymes responsible for the proteolytic activity during leaf senescence [56]. A number of cDNA clones encoding cysteine proteinases in different plants have been previously reported to be up-regulated during leaf senescence [57]. Protein kinases and autophagy-related genes were also reported to play an important role in leaf senescence [58]. 

In the present study, we reported our work on the high-resolution genetic mapping of the novel early leaf senescence gene *Els2*. First, the development of 12 KASP markers from the high-density SNP arrays enabled us to construct high-resolution genetic maps around the target locus. Second, we physically delimited the *Els2* gene to a 1.61-Mb genomic region including 27 putative genes. We believe our study has made a solid foundation for the future isolation of the *Els2* locus.

## 4. Materials and Methods 

### 4.1. Plant Materials and Population Construction

The novel early leaf senescence mutant LF2099 was identified from the chemical mutagen EMS-induced library derived from the common wheat accession H261. To develop a saturated genetic linkage map for *Els2*, the F_2_ and F_2:3_ mapping populations (consisting of 838 lines) produced by crossing LF2099 to the normal leaf senescence cultivar Chinese Spring were used throughout the study. All plants were grown on the experimental farm of Northwest A&F University, Yangling, China. Leaf senescence phenotypes were evaluated by visual observation. To ensure the accuracy of the results, the phenotypes of the recombinant F_2_ individuals were further validated by using the F_3_ lines. The F_3_ lines containing 30 seeds from every F_2_ plant were planted in rows to verify the phenotypes of the F_2_ plants in fields.

### 4.2. Combined Bulked Segregant Analysis Using the 660K SNP Array

The total genomic DNA from the parents and F_2_ plants were isolated from leaves by using a cetyltrimethylammonium bromide (CTAB) method [59]. The DNA quantity was measured spectrophotometrically, and the DNA integrity was confirmed on agarose gel. To obtain closer SNP markers and saturate the targeted gene, BSA was performed to identify polymorphic markers between the early and normal DNA bulks. The DNA of the 12 homozygous plants that exhibited early leaf senescence and normal leaf senescence, based on a validation of the phenotypes of the F_3_ family, was pooled to construct an early bulk sample and a normal bulk sample, separately. The F_2_ bulks were genotyped with the 660K SNP arrays from CapitalBio Corporation (Beijing, China) using the Affymetrix GeneTitan System based on the Axiom 2.0 Assay Manual Workflow protocol. Allele calling was performed using the Affymetrix proprietary software according to Axiom Best Practices Genotyping Workflow. The sequences of all SNPs were used to blast version1.0 of the assembled Chinese Spring survey sequence (International Wheat Genome Consortium (IWGSC) RefSeq v1.0) to determine their physical positions [51]. 

### 4.3. Conversion of SNP Markers to KASP Markers

The polymorphic SNP markers from 90K and 660K were converted to KASP markers using the PolyMarker software (available online: http://polymarker.tgac.ac.uk) [60]. Chromosome-specific KASP markers were used to screen the parents and small population to confirm the polymorphisms before genotyping the entire mapping population. The KASP reactions were performed in a 384-well plate format following the protocol of LGC Genomics. Each KASP reaction mixtures consisted of final volumes of 5 μL containing 50–100 ng of genomic DNA, 2.5 µL of 2 × KASP Master mix (V4.0, LGC Genomics, Hoddesdon, UK), 0.056 µL of assay primer mix (12 µM of each allele-specific primer and 30 µM of common primer) and 2.444 µL of water. The assay reaction was performed in a 384 well Thermal Cycler (Bio-Rad) in the following cycling conditions: denaturation at 95 °C for 15 min, 9 cycles of 95 °C for 20 s and touchdown starting at 65 °C for 60 s (decreased by 0.8 °C per cycle), 30–40 cycles of amplification (95 °C for 20 s; 57 °C for 60 s). The amplified products were visualized by a microplate reader (FLUOstar Omega, BMG LABTECH, Germany) and analyzed by the software Klustering Caller (LGC, Middlesex, UK) [61].

### 4.4. Construction of High-Density Genetic Linkage Map

The linkage analysis of the polymorphic molecular markers and the *Els2*gene was conducted using JoinMap4.0 software [62] with default parameters and a LOD score of 3.0 as a threshold. The genetic distance was calculated and presented in Kosambi centiMorgans (cM). The genetic map was drawn with the software Mapdraw V2.1 (Huazhong Agricultural Sciences, Wuhan, China) [63].

### 4.5. Physical Mapping and Gene Annotation of the *Els2* Region 

All 14 markers used for the fine mapping of the *Els2* were anchored to the wheat reference genome sequence [51]. All sequences including forward and reverse primers were searched against the Chinese Spring whole genome assembly sequences (Reference Sequence v1.0, IWGSC, http://www.wheatgenome.org/) using the BLASTN algorithm applying default parameters. Obtained physical positions of mapped markers were visualized using the software Mapdraw V2.1 (Huazhong Agricultural Sciences, Wuhan, China) [63]. The information on the Chinese Spring genomic sequence was used to identify the genes that were included in the interval between the two *Els2*-flanking markers. IWGSC RefSeq v1.1 with gene annotations became available on the website https://wheat-urgi.versailles.inra.fr/Seq-Repository/Annotation. Annotated genes in the target region were extracted for analyzing genes involved in plant leaf senescence.

## Figures and Tables

**Table 1:** Primer sequences of Kompetitive allele-specific polymerase (KASP) markers developed from the 90K and 660K arrays.

Sources	Marker Name (SNP ID)	Primer Sequences (5’–3’)
90K	BS00030364_51	Forward A_FAM: GAAGGTGACCAAGTTCATGCTACAGCCAAAGACCCATCTTAA
		Forward B_HEX: GAAGGTCGGAGTCAACGGATTACAGCCAAAGACCCATCTTAG
		Common reverse: GTGCGAACAGTTGACAGTGA
90K	BS00084417_51	Forward A_FAM: GAAGGTGACCAAGTTCATGCTGCTGGAACTCGCCTCTTCTA
		Forward B_HEX: GAAGGTCGGAGTCAACGGATTGCTGGAACTCGCCTCTTCTC
		Common reverse: ATTTCAGCCCATCATCCTCC
90K	RFL_Contig4423_529	Forward A_FAM: GAAGGTGACCAAGTTCATGCTCATACTCACAGTAACAGATTGCAT
		Forward B_HEX: GAAGGTCGGAGTCAACGGATTCATACTCACAGTAACAGATTGCAC
		Common reverse: GCTTCTGCCACAAGCTCT
660K	AX-94995576	Forward A_FAM: GAAGGTGACCAAGTTCATGCTCGTGCATCTAAACCCACGTATA
		Forward B_HEX: GAAGGTCGGAGTCAACGGATTCGTGCATCTAAACCCACGTATC
		Common reverse: AGAGTCTCTATACCGTGATCCG
660K	AX-95682571	Forward A_FAM: GAAGGTGACCAAGTTCATGCTATGTGTGGTTCCATTCACCG
		Forward B_HEX: GAAGGTCGGAGTCAACGGATTATGTGTGGTTCCATTCACCA
		Common reverse: CAAGTCTGAGTGGAAGAAGGAC
660K	AX-111595417	Forward A_FAM: GAAGGTGACCAAGTTCATGCTTTCAGAAGAAATAGATGTATGTGCC
		Forward B_HEX: GAAGGTCGGAGTCAACGGATTTTCAGAAGAAATAGATGTATGTGCA
		Common reverse: CCATCCCATAAACCCAGGG
660K	AX-94859941	Forward A_FAM: GAAGGTGACCAAGTTCATGCTCCTCAGCTTTCTTTAGGGCT
		Forward B_HEX: GAAGGTCGGAGTCAACGGATTCCTCAGCTTTCTTTAGGGCC
		Common reverse: AATTCCGGTTCACTGGTCCA
660K	AX-111600462	Forward A_FAM: GAAGGTGACCAAGTTCATGCTCCAAAAATAAATTCCGCTAGTCCT
		Forward B_HEX: GAAGGTCGGAGTCAACGGATTCCAAAAATAAATTCCGCTAGTCCC
		Common reverse: CCAAAAACCCAATTGCAAGGTTAA
660K	AX-111497410	Forward A_FAM: GAAGGTGACCAAGTTCATGCTGAATGCAAAGGTTGGCCCT
		Forward B_HEX: GAAGGTCGGAGTCAACGGATTGAATGCAAAGGTTGGCCCG
		Common reverse: CTCGTGCGTACCTGCTCATT
660K	AX-109407129	Forward A_FAM: GAAGGTGACCAAGTTCATGCTTCTTGCCACCGTTTCAATAGAT
		Forward B_HEX: GAAGGTCGGAGTCAACGGATTTCTTGCCACCGTTTCAATAGAC
		Common reverse: CTTTTCTTGGTCTATGTAGTTGGG
660K	AX-111128667	Forward A_FAM: GAAGGTGACCAAGTTCATGCTGCGGGCAAAGCTTTGGATG
		Forward B_HEX: GAAGGTCGGAGTCAACGGATTGCGGGCAAAGCTTTGGATA
		Common reverse: GAGGTAGCGACGATGTCCC
660K	AX-111643885	Forward A_FAM: GAAGGTGACCAAGTTCATGCTGATGCAAATCCAAACCCATTCT
		Forward B_HEX: GAAGGTCGGAGTCAACGGATTGATGCAAATCCAAACCCATTCG
		Common reverse: GCAGGAGGAGAGCTTGGGA

**Table 2:** Gene models between the two flanking markers AX-111643885 and AX-111128667 based on the International Wheat Genome Consortium (IWGSC) RefSeq v1.1 annotation.

Gene	Annotation
TraesCS2B02G575700.1	DEAD-box ATP-dependent RNA helicase 52A
TraesCS2B02G575800.1	CC-NBS-LRR family disease resistance protein
TraesCS2B02G575900.1	50S ribosomal protein L14
TraesCS2B02G576000.2	OTU domain-containing protein, putative
TraesCS2B02G576100.2	Calreticulin-like protein
TraesCS2B02G576200.1	cysteine-rich RLK (RECEPTOR-like protein kinase) 29
TraesCS2B02G576300.1	Cysteine proteinase
TraesCS2B02G576400.1	Serine/threonine-protein kinase
TraesCS2B02G576500.1	Myb family transcription factor family protein
TraesCS2B02G576600.2	lectin-receptor kinase
TraesCS2B02G576700.1	Cytochrome P450
TraesCS2B02G576800.1	RING/U-box superfamily protein
TraesCS2B02G576900.1	Chromosome segregation in meiosis protein 3
TraesCS2B02G577000.1	RING/U-box superfamily protein
TraesCS2B02G577100.1	Cytochrome P450 family protein, expressed
TraesCS2B02G577200.1	Cytochrome P450 family protein, expressed
TraesCS2B02G577300.1	Cysteine proteinase
TraesCS2B02G577400.1	Cytochrome P450 family protein, expressed
TraesCS2B02G577500.1	Cysteine proteinase
TraesCS2B02G577600.1	Serine/threonine-protein phosphatase 7 long form-like protein
TraesCS2B02G577700.1	Phytol kinase 1
TraesCS2B02G577800.1	Exostosin family protein
TraesCS2B02G577900.1	B3 domain-containing protein
TraesCS2B02G578000.1	Polyadenylate-binding protein 1-B-binding protein
TraesCS2B02G578100.1	ATP-dependent Clp protease proteolytic subunit 3
TraesCS2B02G578200.4	Serine/threonine-protein kinase
TraesCS2B02G578300.1	Cytochrome P450, putative
